# Challenges in the design, planning and implementation of trials evaluating group interventions

**DOI:** 10.1186/s13063-019-3807-4

**Published:** 2020-01-29

**Authors:** Katie Biggs, Daniel Hind, Rebecca Gossage-Worrall, Kirsty Sprange, David White, Jessica Wright, Robin Chatters, Katherine Berry, Diana Papaioannou, Mike Bradburn, Stephen J. Walters, Cindy Cooper

**Affiliations:** 1 School of Health and Related Research (ScHARR) University of Sheffield, Regent Court, 30 Regent Street, Sheffield, S1 4DA UK; 2 Nottingham Clinical Trials Unit (NCTU), University of Nottingham, Nottingham, UK; 3 School of Health Sciences, University of Manchester, Manchester, UK

**Keywords:** Group interventions, Therapy groups, Treatment fidelity, Implementation, Intervention design, Clinical trials

## Abstract

**Background:**

Group interventions are interventions delivered to groups of people rather than to individuals and are used in healthcare for mental health recovery, behaviour change, peer support, self-management and/or health education. Evaluating group interventions in randomised controlled trials (RCTs) presents trialists with a set of practical problems, which are not present in RCTs of one-to-one interventions and which may not be immediately obvious.

**Methods:**

Case-based approach summarising Sheffield trials unit’s experience in the design and implementation of five group interventions. We reviewed participant recruitment and attrition, facilitator training and attrition, attendance at the group sessions, group size and fidelity aspects across five RCTs.

**Results:**

Median recruitment across the five trials was 3.2 (range 1.7–21.0) participants per site per month. Group intervention trials involve a delay in starting the intervention for some participants, until sufficient numbers are available to start a group. There was no evidence that the timing of consent, relative to randomisation, affected post-randomisation attrition which was a matter of concern for all trial teams. Group facilitator attrition was common in studies where facilitators were employed by the health system rather than the by the grant holder and led to the early closure of one trial; research sites responded by training ‘back-up’ and new facilitators. Trials specified that participants had to attend a median of 62.5% (range 16.7%–80%) of sessions, in order to receive a ‘therapeutic dose’; a median of 76.7% (range 42.9%–97.8%) received a therapeutic dose. Across the five trials, 75.3% of all sessions went ahead without the pre-specified ideal group size. A variety of methods were used to assess the fidelity of group interventions at a group and individual level across the five trials.

**Conclusion:**

This is the first paper to provide an empirical basis for planning group intervention trials. Investigators should expect delays/difficulties in recruiting groups of the optimal size, plan for both facilitator and participant attrition, and consider how group attendance and group size affects treatment fidelity.

**Trial registration:**

ISRCTN17993825 registered on 11/10/2016, ISRCTN28645428 registered on 11/04/2012, ISRCTN61215213 registered on 11/05/2011, ISRCTN67209155 registered on 22/03/2012, ISRCTN19447796 registered on 20/03/2014.

## Included trials

JtD [1] Journeying through Dementia.

LM [2] Lifestyle Matters.

PLINY [3] Putting Life IN Years.

REPOSE [4] Relative Effectiveness of Pumps Over Structured Education.

STEPWISE [5] STructured lifestyle Education for People WIth SchizophrEnia.

## Background

### Group interventions in healthcare

Group interventions are used as an alternative, or in addition to, interventions delivered to individuals in healthcare [6, 7] and involve an intervention delivered to small groups of people by one or more group leaders rather than to individuals; this includes activity, support, problem solving/educational and psychodynamic groups, but does not includes task or work groups or large education groups [8]. Originally focusing on mental health recovery [6], they now often also focus on behaviour change, peer support, self-management and/or health education [7].

Group interventions can present opportunities for costs savings by treating more than one person at the same time. In addition, advocates of group interventions have proposed mechanisms of action that are important for behaviour change that arise from being in a group that are not present in individual therapies, such as inter-personal change processes, universalisation, social comparison, social learning and modelling [6, 7, 9, 10]. The role of group process and dynamics in these mechanisms is contested, with some believing that these mechanisms of action can be triggered by individual–therapist interaction [11] and others proposing that the group aspect is an essential part of the intervention [12].

Mixed evidence exists for the effectiveness of group interventions. Group interventions improve health outcomes compared to individual therapy in smoking cessation [13], breastfeeding [14] and weight management [15, 16]; compared to usual care or no intervention in diabetes [17]; and, are equally effective as individual therapy in obsessive-compulsive disorder [18].

Clinically effective group interventions do not always lead to anticipated cost savings compared to individual treatments, with trade-offs between numbers of patients treated and the duration or quality of the programmes [19, 20]. Compared with an individual modality, cognitive behavioural therapy for insomnia [21] and weight management [15] groups were found to be cost-effective, whereas smoking cessation groups were not [13]. Particularly in mental health, there is some concern that the cost-effectiveness of group interventions compares poorly with one-to-one therapy [22–26]. It is also said that certain populations may not be suited to group therapy, including those with communication problems, disruptive behaviour or co-morbidities that make it hard to relate to other group members [25].

Group interventions in healthcare tend to be small groups which involve interaction between members [8]. Small groups are said to move through five stages: the establishment of ground rules; conflict; cohesion; structure supportive of task performance; and, termination [7, 27, 28]. This staged development is sometimes used as an argument for closing group membership after initial sessions, notwithstanding member attrition, which is common [29]. Optimal group size for group interventions is said to depend on the type and duration of therapy, as well as the target population. There is consensus that ideal group size is 7–8 members, with a range of 5–10 members [6, 30–33]. Groups with five or more members allow the formation of meaningful relationships [34] and cohesive group functioning [6]. Although some maintain that therapeutic benefit can be derived in groups with < 5 members [35, 36], there is evidence that with < 5 members, interaction, group identity, attendance and group image is poor [6, 37]. Upper limits to group size may depend on how many people a therapist can practically manage [38] but it has been found that fewer verbal interrelationships occur [33] in groups with > 8 members, and social fission [39] and conflict [40] are more common in larger groups.

### Evaluation of group interventions

In addition to well-documented statistical concerns around therapist effects and clustering [41], a number of approaches to evaluating group interventions have been proposed. Recognising that the design, evaluation and reporting of group interventions require additional information to that which is routinely collated for individual interventions, Hoddinott and colleagues developed a framework [19] to supplement the Medical Research Council (MRC) guidance on complex interventions [42]. For instance, in addition to the intervention content and theory, which would be the same in one-to-one delivery, documentation of group membership and maintenance processes (planning, setting up, organising and sustaining the group), as well as well as the leader/member attributes are pivotal to understanding how the intervention works. Borek and colleagues developed a checklist for the reporting of group-based behaviour change interventions and a framework detailing the mechanisms of action for group interventions, which helps researchers describe intervention design and content, participants and facilitators, and to determine the mechanisms of action present in group interventions [10, 43].

This paper is intended as a supplement to these developments and outlines practical challenges to the implementation of group-based therapies in randomised controlled trials (RCTs). The data provide a ‘reference-class’ – data from past, similar projects which can be used for forecasting [44]. Researchers can use reference class data to plan and manage trials as well as forecast contingencies related to: participant recruitment, randomisation and attrition; the demand and supply aspects of intervention delivery; therapeutic dose; group size; and process evaluation.

The aim of the present paper is to provide practical guidance to the implementation of group-based intervention randomised trials based on previous experience of five group intervention trials conducted by the Sheffield Clinical Trial Research Unit (CTRU).

## Objectives

The primary objective is to present reference class data specific to group intervention trials on participant recruitment and attrition, facilitator training and attrition, group attendance, therapeutic dose and group size.

The secondary objectives are to provide explanations and potential solutions for problems observed in group intervention trials which are substantively different to those observed in studies of individual-level interventions.

## Methods

### Case studies

A case-based approached was adopted to present the challenges of implementing group interventions in five RCTs [1–5] evaluating group interventions (Table 1) managed by Sheffield CTRU [45]—a UK Clinical Research Collaboration (UKCRC)-registered clinical trial unit managing phase III RCTs of a range of interventions across varied research areas. The CTRU has managed a number of evaluations of complex interventions, including five completed group intervention trials.

**Table 1:** Details of case studies

Study	Population (target)	Group intervention [additional sessions]	Facilitator (per session)	Method and timing of recruitment	Type and timing of randomisation	Outcome data collection
LM [2]	65+ years (prevention: general)	16 × weekly sessions: 2-h face-to-face occupational therapy [4 × one-to-one sessions]	Two NHS Band 4 equivalent staff. Recruited on university contracts specifically to deliver the intervention Training: two- day face-to-face course	Mail-out via GPs, healthcare referral and self-referral from study promotion (including researchers visiting dementia cafes and other groups)	Individual. Done by central team immediately after consent and baseline data collection	Central research assistants; blinded outcome assessor; follow-up anchored to randomisation
PLINY [3]	75+ years (prevention: loneliness)	12 × weekly sessions: 1-h telephone friendship [6 × one-to-one telephone calls before group]	One volunteer from a community organisation. Training: 4 × 1 h sessions via telephone	Mail-out via GPs and to research cohort	Individual. Done by central team immediately after consent and baseline data collection	Central research assistants; blinded outcome assessor; follow-up anchored to randomisation
REPOSE [4]	18+ years. Type I diabetes (therapy: self-care education)	5 × daily sessions: full-day face-to-face Education (total approx. 38 h). [1 optional × group follow-up session]	Two diabetes specialist nurses/dieticians. Training: five- day observation, three-day face-to-face workshop, peer-reviewed delivery of five-day course and one-day workshop (105 h)	Referral via care team in person or via mail-out	Cluster by course in pairs. Delayed randomisation: after groups were filled, 6 weeks before first course; baseline taken after randomisation	Facilitator at clinic visits; unblinded outcome assessor; follow-up anchored to group attendance
STEPWISE [5]	18+ years. First episode psychosis + schizophrenia (prevention: cardiovascular)	4 × weekly sessions: 2.5-h face-to-face. [3 × quarterly booster group sessions and fortnightly 1:1 support calls between booster sessions]	Two NHS staff (mental health staff; occupation therapists and dieticians). Training: three-day face-to-face course plus one-day booster session training	Referral via care team	Individual. Done immediately after consent and baseline data collection	CMHT staff or research nurses; blinded outcome assessor; follow-up anchored to randomisation
JtD [1]	18+ years. Dementia (prevention: dependency)	12 × weekly sessions: Approx. 2-h face-to-face psychosocial education [4 × one-to-one sessions]	Two NHS staff. Training: two-day face-to-face course, plus online resources	Mail-out via GPs/care teams, mail-out to research cohort by research team, referral via care team, or self-referral from study promotion (including researchers visiting dementia cafes and other groups)	Individual. Delayed randomisation: after collection of baseline data < 2 months before intervention	Central and local site research assistants; blinded outcome assessor; follow-up anchored to randomisation

*GP* general practitioner

Data were collated from trial reports and journal articles, from the trial data held in Sheffield CTRU and from the study managers; descriptive statistics are presented.

Of the included trials, one was cluster-randomised [4] and all others were individually randomised. Lifestyle Matters [2] (LM) was a two-centre trial assessing a psychosocial group intervention to promote healthy ageing in adults aged ≥ 65 years with reasonable cognition. Putting Life IN Years [3] (PLINY) was a single-centre RCT that aimed to evaluate a group telephone-befriending intervention to prevent loneliness in adults aged ≥ 75 years with reasonable cognition. Relative Effectiveness of Pumps Over Structured Education [4] (REPOSE) was an eight-centre cluster RCT assessing an existing group educational course for use with multiple daily injections compared to the same intervention adapted for use with a pump for adults aged ≥ 18 years with type 1 diabetes. The STructured lifestyle Education for People WIth SchizophrEnia [5] (STEPWISE) RCT ran in 10 mental health organisations and evaluated a group structured weight management lifestyle education intervention in adults aged ≥ 18 years with schizophrenia, schizoaffective disorder or first episode psychosis. Journeying through Dementia [1] (JtD) was a 13-centre RCT assessing a group intervention designed to support people in the early stages of dementia to maintain independence. All trials took place in the UK.

Various methods for recruitment were used in these trials and some studies used more than one method [1–3], including: mail-outs via general practitioners (GPs)/NHS care teams [1–4]; mail-outs to the research cohort [1, 3]; referrals via NHS care teams [1, 4, 5]; and self-referral [1, 2].

Individual randomisation was used in four of the trials [1–3, 5] and cluster randomisation [4] was used in one. Randomisation was delayed from the point of consent in two trials [1, 4] to ensure that the groups were filled and could be run in the time frame required. Follow-up data collection was anchored to the time of randomisation in four of the trials [1–3, 5] and to the commencement of the first group in one trial [4].

All groups ran for more than one session: one group intervention [4] took place on five consecutive days, all other included studies had weekly sessions in the range of 4–16 weeks and all of the studies had additional sessions to the main group intervention. All included interventions were face-to-face sessions, except for one which was a telephone-befriending group [3]. A variety of people facilitated the group sessions in the trials such as NHS staff [1, 2, 4, 5] and volunteers [3]; all received structured training in the group intervention and collected research data in relation to the attendance at group sessions. At least two facilitators delivered all of the face-to-face interventions and one person delivered the intervention via telephone in PLINY [3].

All included studies used some aspect of treatment fidelity assessment: direct observation [1, 4, 5] or recording [2, 3] of a session using a checklist; self-report by facilitators using a checklist [1] in addition to observation; and assessment of facilitator–participant interaction [5]. In addition, training fidelity was assessed in three trials by two researchers either by direct observation [1, 2] or using audio recordings [3] of training sessions.

Many of the elements discussed above are relevant to RCTs in general and to RCTs of complex interventions but some need particular consideration in relation to group interventions. The type and timing of recruitment and randomisation are particularly important as these will dictate when the group sessions can be arranged and how much time there is to train facilitators. Practical arrangements for group sessions will be affected by the population [46], group size, type and length of training, the mode of group delivery and who the facilitator is.

## Results

### Participant recruitment and attrition

Table 2 shows the number of individuals approached and recruited for each trial. Four studies recorded data on the numbers invited to screen for eligibility and the associated response rate: 4.1% (LM [2]); 2.9% (PLINY [3]); 69.2% (REPOSE [4]); and 7.1% (JtD [1]). In REPOSE [4], acute care teams targeted people with type 1 diabetes, compared with the other studies in which GPs sent out mass mail-outs. LM [2], PLINY [3] and STEPWISE [5] were also prevention trials rather than treatment trials, which have shown to be harder to recruit to [47]. The proportion of those screened providing consent is higher for trials using initial GP mass mail-outs than for other trials; it is lowest in STEPWISE [5], which recruited participants with schizophrenia which can be a difficult population to recruit to trials [48].

**Table 2:** CONSORT data

	Invited to take part (mail-out only)	Response rate to mail out (% of sent)	Screened	Eligible (% of screened)	Consented (% of screened)	Randomised (% of screened)	Attended at least one group session (% of randomised)
Randomisation concurrent with consent							
LM [2]	9330 ^a^	385 (4.1) ^a^	313	294 (93.9)	288 (92.0)	288 (92.0)	123/145 (84.8)
PLINY [3]	9579 ^b^	275 (2.9) ^b^	178	159 (89.3)	157 (88.2)	157 (88.2)	21/78 (26.9)
STEPWISE [5]	N/A	N/A	1223	989 (80.9)	423 (34.6)	414 (33.9)	171/208 (82.2)
Delayed randomisation							
REPOSE [4]	1278	885 (69.2)	362	334 (92.3)	321 (88.7)	317 (87.6)	267/317 ^c^ (84.2)
JtD [1]	958 ^a^	68 (7.1) ^a^	1183	521 (44.1)	520 (43.9)	480 (40.6)	217/239 (90.8)

*GP* general practitioner

^a^ Numbers relate to GP mail outs only

^b^ Numbers relate to GP and research cohort mail-out only

^c^ Participants allocated to both the intervention and control arms attended group sessions

### Setting group dates

The trials had different approaches to setting the days and times for the group sessions. Due to the intervention being used outside of the trial, REPOSE set the dates in advance of participant recruitment, patients knew when the groups were at the time of consent and the courses were randomised once the required numbers were met (usually a minimum of five participants per group). LM [2] set provisional dates or windows for the group sessions but finalised the times and dates with the participants once group numbers were met. STEPWISE [4] asked sites to block book consent visits (where practical) and to set course dates in advance which delayed consent for some participants; sites decided how they would implement this. The purpose was to minimise post-randomisation attrition, ensure follow-up occurred after intervention delivery and to optimise group size. JtD [5] commenced without pre-planning the dates for the intervention but as the trial progressed, the trial team advised sites to set the dates before consent and many did so. Although these dates sometimes changed, the trial team ensured that any moved dates were on the same time and day of the week to increase the possibility of attendance. PLINY [3] did not pre-plan timing for the groups and relied on the service provider to set the date once the group had been recruited. As only one trial explicitly set the dates before randomisation, we cannot explore the impact of these differences in our data.

### Attrition

Attrition of participants between consent and randomisation occurred where randomisation was delayed, as can be seen in the data for REPOSE [4] (*n* = 4) and JtD [1] (*n* = 40). Although randomisation was not delayed in STEPWISE, there is some attrition between consent and randomisation (*n* = 9). Reasons for this were withdrawal of consent (*n* = 4), mental health deterioration (*n* = 4) and surgery (*n* = 1), which suggests that there was a delay in randomising after consent [5], though it was not designed this way. The percentage of those attending at least one group session appears unaffected by the timing of randomisation or by when the days and times of the group sessions were set.

We have found that maintaining contact with participants between any of these stages can reduce attrition while they are waiting for randomisation or for group sessions to be arranged [49, 50]. In LM, once randomised, facilitators contacted the participants allocated to the intervention arm to introduce themselves and start discussing possible dates/times for the next group meeting. The participant would then be aware of timings including how long it might be to get a group started; they would also arrange the first one-to-one session with the participant to start relationship building. The facilitators maintained this contact while waiting for the group intervention to start. Another challenge that arose from delayed randomisation related to follow-up: when groups of people were randomised at the same time and follow-up was anchored to randomisation, all of the group members needed to be followed up at the same time point.

Table 3 shows the recruitment rate by site and by month for each trial; this is a crude estimate as we have assumed all sites were open for the whole recruitment period, which is rarely the case. The median (range) recruitment rate for all included studies is 3.2 (1.7–21.0) participants per site per month.

**Table 3:** Recruitment rates

	Total randomised	Total months recruiting	Number of recruiting sites	Recruitment rate (total randomised/site/month)
LM [2]	288	8.3 ^a^	2	17.4
PLINY [3]	157	7.5 ^b^	1	21.0
REPOSE [4]	317	16.7 ^b^	8	2.4
STEPWISE [5]	414	12.9 ^a^	10	3.2
JtD [1]	480	21.2 ^a^	13	1.7

Start date of recruitment taken as either: ^a^ the date of the first randomised participant; or ^b^ the 1st of the month (where the starting month is reported). The end date is the last randomised participant in all cases

### Participant demand and facilitator supply

With group interventions, the planned (and actual) recruitment rate needs to be linked to the delivery of the intervention so that enough people are randomised to a group without having to wait too long to start the sessions in order to reduce attrition. This should be forecast in the early stages of RCT design to ensure an accurate schedule for the whole trial, taking into account facilitator training, room booking and other practical aspects of delivery. Training varied in intensity (See Table 1 for details), with the training for REPOSE [4] being the most intensive although, unlike in other trials, facilitators were trained before and independently of the research programme.

### Facilitator training

Attrition and replacement of trained facilitators should be anticipated. Apart from LM [2], studies where facilitators were trained solely for the research had some attrition of facilitators and both STEPWISE and JtD had to run more training sessions than had initially been planned for the trial. Although LM [2] did not experience facilitator attrition, one of the facilitators had a period of sick leave and their sessions were covered by the chief investigator and another person who required facilitator training. Recruitment of facilitators can also present difficulties. In JtD [1], the facilitators were supposed to be provided by the trust, but they often filled these roles with NHS R&D staff as other staff could not be recruited to fill the roles. PLINY [3] did not manage to recruit the required number of volunteers to deliver the intervention (Table 4).

**Table 4:** Facilitator training and delivery

	Total number of facilitators fully trained	Number of training sessions - actual (planned)	Number delivering at least one group session
LM [2]	5	2 (2)	6 ^a^
PLINY [3]	10	3 (6)	3
STEPWISE [5]	44 ^b^	6 (4)	14
JtD [1]	69 ^c^	12 (4)	60

^a^ Includes the Chief Investigator who did not require training as they developed the intervention

^b^ Foundation training and booster training was delivered separately; here we only include those attending both courses

^c^ Supervisors were also trained in the intervention and have not been included in these numbers

Note: REPOSE [4] is not included in this table as the course ran outside of the trial and therefore the facilitators were trained and managed outside of the research team

#### PLINY case study: facilitator supply did not meet participant demand

The PLINY [3] trial had to be stopped prematurely as there were not enough facilitators to deliver the intervention. PLINY [3] and the service providers (facilitators) planned to have seven groups of at least six participants, with staggered start dates so that all groups were running concurrently by week 16. The start of recruitment was delayed from May 2012 to June 2012 and an increased mail-out was required in October 2012 in order to achieve the recruitment target. This successful recruitment strategy meant there were randomised participants (demand) that required group sessions to be delivered (supply); in this case, supply did not match the demand.

PLINY [3] was particularly vulnerable to poor supply–demand matching. Funding for the training and hosting of facilitators sat outside of the University research team, as demanded by the excess treatment cost system – a peculiarity of UK NHS R&D funding [51–55]. Notwithstanding contractual obligations to a research project, if a service provider has other priorities, the research team have little leverage. In LM [2] and other trials where facilitators were funded through research grants and employed by the research project, we have observed efficient supply–demand profiles, despite the common problems in participant recruitment.

**Fig. 1:**
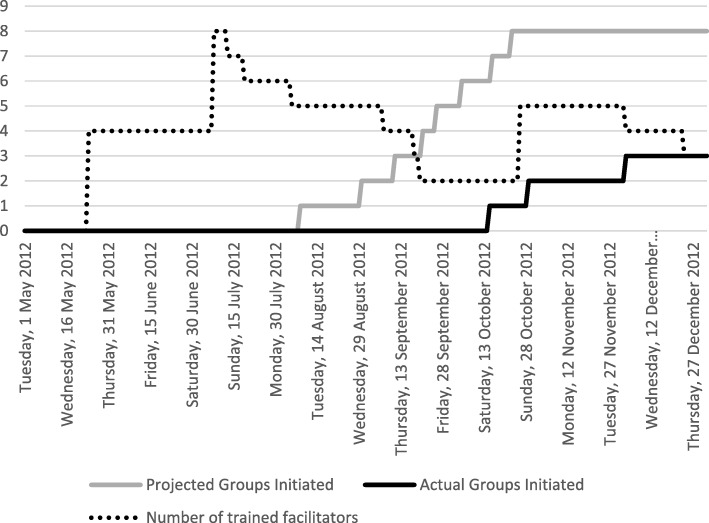
Participant demand, supply of facilitators and group delivery graph for PLINY

Figure 1 shows the availability of facilitators against the demand for group sessions. Experienced volunteer coordinators provided induction and supervision, and an experienced external trainer provided formal group facilitation training to facilitators so that the group intervention could be delivered to the target number of participants (*n* = 124). Funding was secured from a national charity to do this, which meant that only local branches of their charity could deliver the intervention, rather than a number of service providers originally planned. Recruitment, training and supervision of facilitators was therefore the community organisation’s contracted responsibility and they were in close contact with the trial team and were informed of participant recruitment numbers during the trial. Out of the 42 volunteers that expressed an interest in delivering the group intervention, 10 completed the training and only three delivered the group sessions; the mean time a volunteer stayed with the project after they had been trained was 62 days (range 12–118).

### Therapeutic dose

The ‘therapeutic dose’ necessary for a change to occur in complex interventions may be related to certain criteria being delivered rather than the number of sessions attended [56]. However, a ‘therapeutic dose’ relating to attendance is often agreed upon in trials to define the per-protocol population. In our experience, this has been decided through consensus of the trial management groups and the trial steering committees for each trial. Table 5 shows that the ‘therapeutic dose’ in our trials was an attendance rate in the range of 28.6%–80% of the planned sessions.

**Table 5:** Number of sessions attended and numbers achieving therapeutic dose

	Minimum number of sessions for ‘therapeutic dose’ (or per protocol population)	Median (range) sessions attended	Number of participants achieving ‘therapeutic dose’ (% of those receiving any intervention)	Number attending all planned sessions (% of those receiving any intervention)
LM [2]	12/16 (75%)	12 (1–16)	71/123 (57.7%)	5/123 (4.1%)
PLINY [3]	9/12 (75%)	11 (2–12)	9/21 (42.9%)	10/21 (47.6%)
REPOSE [4]	4/5 (80%)	5 (1–5)	261/267 (97.8%)	250/267 (93.6%)
STEPWISE [5]	2/7 ^a^ (28.6%)	Foundation: 3 (0–4) Booster: 2 (0–3)	161/171 ^a^ (94.2%)	47/171 ^a^ (27.5%)
JtD [1]	10/16 ^b^ (62.5%)	1:1 sessions: 4 (1–4) Groups: 9 (0–12)	168/217 ^b^ (77.4%)	36/217 ^b^ (16.6%)

^a^ Foundation and booster sessions included

^b^ Group and 1:1 sessions included

Across five group therapy programmes, the median percentage of participants receiving a ‘therapeutic dose’ was 76.7% (range 42.9%–97.8%). REPOSE [4], a treatment trial, where the course ran on five consecutive days was the most successful at achieving the defined therapeutic dose (97.8%) and also achieving attendance at all sessions (93.6%). Participant motivation to attend group interventions may be related to the motivation to enrol in research and therefore may be higher for treatment trials than for prevention trials [47]. However, JtD, a treatment trial, does not achieve the high ‘therapeutic doses’ of REPOSE and STEPWISE, and only REPOSE had > 50% of participants attending all sessions. In addition, participants usually had to take a week off work to ensure attendance at all group sessions for REPOSE [4]. For groups that ran weekly for several weeks, availability may have been more difficult and the time in between sessions may have led to a change in motivation or willingness to attend. This can be seen in STEPWISE as total attendance at the group sessions reduced each week (144 participants attended their week 1 session, 138 participants attended weeks 2 and 3, and 131 participants attended week 4). Booster sessions were 4, 7 and 10 months after randomisation and had fewer attendees than the foundation group sessions (100, 89 and 90, respectively).

### Group size

Table 6 presents the ideal and actual group sizes for each group intervention.

**Table 6:** Group sizes

	Ideal group size	Mean number of participants per group (SD) ^a^	Median number of participants per group (IQR) ^a^	Minimum and maximum group size ^a^	Number of sessions delivered with correct group size (% of number of sessions delivered ^a^)	Number of sessions with 0 or 1 person recorded (% of planned sessions)
LM [2]	8–16	7.7 (2.42)	7 (6–9)	2–15	80/173 (46.2%)	13/186 (6.9%)
PLINY [3]	6–8	5.1 (1.17)	6 (4–6)	3–6	24/41 (58.5%)	1/42 (2.4%)
REPOSE [4]	5–8	5.8 (1.38)	6 (5–7)	3–8	36/46 (78.3%)	0/46 (0%)
STEPWISE [5]	6–8	Foundation: 4.2 (1.69)	4 (3–5)	2–9	25/129 (18.9%)	3/132 (2.3%)
		Booster: 3.5 (1.41)	3 (2–4)	2–8	7/75 (7.1%)	23/98 (23.5%)
JtD [1]	8–12	5.3 (1.71)	6 (4–6)	2–9	32/331 (9.7%)	5/336 (1.5%)

^a^ Sessions recorded in the data with 0 or 1 individual attending have not been included here as this does not constitute a group session, number of sessions where 0 or 1 were recorded are provided in the final column

A total of 45 of 840 (5.3%) planned sessions could not go ahead as only 1 or 0 participants turned up to the session; therefore, a group session could not be delivered. All studies have run groups outside of the ideal range identified for their intervention, with the majority of sessions running with fewer than the ideal numbers (619/826 sessions, 74.9%); STEPWISE [5] ran some groups with more than the ideal numbers (3/826 sessions, 0.4%). REPOSE [4] achieved the ideal group size in 78.3% of cases whereas all other trials managed to achieve the desired group size in < 60% of sessions (median 33.4%). In addition to being a treatment trial that ran daily for one week, REPOSE [4] delayed randomisation until there were sufficient numbers to meet the required group size and, in the early stages, allowed non-participants to join the usual care arm to maintain group size and dynamics. When one group was too small in JtD [1], they allowed additional participants to join the group for the second session so that the ideal group size was met. All included studies involved the monitoring of metrics, such as recruitment and attrition, and intervention adherence there was the opportunity to ensure the ideal group size, for example by combining small groups or adding new members, but only one trial team opted for the addition of new members. In our experience, investigators are often reluctant to add new members to group interventions after initiation as it may affect the group dynamics, and if the intervention is time-limited, it would mean new participants do not have the opportunity to receive the whole course.

### Process evaluation

Process evaluations are often conducted in trials of complex interventions in order to find out what (if any) elements of the intervention are effective, in what circumstances and to whom [57, 58]. For group interventions, the process evaluation should determine if and why people respond differently to the same group sessions. Process evaluation has a number of components: context; reach; dose delivered; dose received; fidelity; implementation; and recruitment [57]—which can all impact on the effectiveness of the intervention. Four of our trials [1–3, 5] included a formal process evaluation based on these fidelity components and also used the MRC framework on the evaluation of complex interventions [42]; three of these trials [2, 3, 5] were designed before the publication of the MRC Process Evaluation Guidance [58]. All trials collected data on the trial population, which provides data relating to reach and recruitment but only three trials used these data a part of a formal process evaluation. LM found that the intervention was delivered correctly and was tailored to groups but reach and recruitment were issues that led to the intervention not being effective as the participants may not have been at a stage where the intervention would have helped them. STEPWISE found reach and recruitment to be acceptable but fidelity to the intervention was incomplete. As previously discussed, PLINY [3] experienced issues with implementation due to facilitator attrition which relates to reach, dose delivered and dose received, but the fidelity assessments also identified issues with delivery and receipt of treatment.

Table 7 details the fidelity strategies and assessments used in the trials, apart from in relation to *design*, as all five trials fully described the interventions in the protocol, including the programme theory where applicable. The programme theory determines the important aspects for the process evaluation and, for group interventions, will include group specific processes. All trials standardised training and intervention materials as a strategy for training fidelity. All trials assessed fidelity in relation to treatment using checklists at a group rather than an individual level using checklists to determine what was delivered by the facilitator. These assessed the delivery of the intervention to the whole group and whether the members took part as intended. The fidelity checklists often included questions asking if the group leader was able to facilitate group processes such as peer exchange, mutual support, group cohesion, group engagement and group goals.

**Table 7:** Fidelity elements included in the trials [59]

	Training	Delivery of treatment	Receipt of treatment	Enactment of skills	Findings ^a^
LM [2]	Standardised training and materials; direct observation of training, checklist used by two researchers	Visual recording of a purposive sample of sessions; assessed by two people using delivery checklist; facilitator supervision; attendance registers	Assessed through qualitative research with a subset of participants at 6 months after randomisation; attendance registers	Assessed through qualitative research with a subset of participants at 6 and 24 months after randomisation	Reported as categorical for fidelity: *Satisfactory (based on a continuous fidelity score of 61%–70%)* in 7/8 recordings for both participant and facilitator performance
PLINY [3]	Standardised training and materials; audio recording of purposive sample of training sessions; checklist used by two researchers; checklist for facilitator skill acquisition	Audio recording of sample of groups at three timepoints; assessed by two people using delivery checklist; facilitator supervision; attendance registers	Checklist for treatment included elements of receipt and assessed through qualitative research with a subset of participants; attendance registers	As for receipt, as enactment of skills should have taken place in the group sessions in this intervention	Reported *percentage scores* for fidelity: Training scores were high but overall fidelity scores for facilitators were low
REPOSE [4]	Standardised training and materials	Direct observation of particular sessions in intervention arm; existing quality assurance for standard DAFNE course; facilitator supervision + feedback from fidelity assessment; attendance registers	Assessed through qualitative research with a subset of participants immediately after intervention; attendance registers	Assessed through qualitative research with a subset of participants at 6 months after intervention	Achievement of essential outcomes assessed as: *Yes*, *Partial* (with a percentage) or *Not Observed*; appear to be delivered as planned; fed back to facilitators
STEPWISE [5]	Standardised training and materials	Direct observation of a sample of 30 sessions in 10 sites using a checklist and an interaction observation tool; also explored in qualitative interviews with a subset of participants, facilitators and intervention developers; facilitator supervision; attendance registers	Assessed through qualitative interviews with a subset of participants and facilitators; checklist for session included elements of receipt and enactment; attendance registers		Reported *percent of facilitator talk time* and the *percent of time* in *positive* and *negative behaviours*; poor intervention fidelity reported
JtD [1]	Standardised training and materials; direct observation of training, checklist used by two researchers; checklist for facilitator skill acquisition	Direct observation of two sessions of four groups at four sites; checklist used by two independent observers; self-report checklist by facilitator at each session; facilitator supervision and supervision checklists; attendance registers	Assessed through qualitative interviews with a subset of participants; attendance registers	Assessed through qualitative interviews with a subset of participants	The fidelity data have not yet been analysed or interpreted

^a^ Findings relating to attendance (delivery and receipt elements) are reported in Tables 5 and 6

STEPWISE [5] used an observation tool during direct observation of sessions to assess a group specific process—the interaction between the facilitator and the participants, as this was considered a key component of the group intervention. The checklists used for assessing treatment delivery fidelity for STEPWISE [5] also included elements relating to the receipt of the intervention and enactment of skills while in the group session.

All included trials conducted some qualitative research that covered acceptability or satisfaction for a subset of participants and facilitators; STEPWISE [5] also explored implementation using Normalisation Process Theory (NPT) [59] and interviewed the intervention developers to inform the process evaluation. In addition, all studies used the qualitative research undertaken with participants to assess fidelity in terms of the receipt of the intervention, with LM [2], REPOSE [4], STEPWISE [5] and JtD [1] also looking at enactment of skills.

### Clustering concerns

#### Couple recruitment

LM [2] recruited 18 couples which presented the study team with issues that are not well documented in the literature, though statistical concerns regarding the analysis of group interventions, or clusters, are well documented [60–64]. In LM [2], couples were randomised as a pair so that they received the same allocation, which reduces the risk of contamination between arms, and is often preferred by paired participants [65]. If couples (or twins) are randomised to the same group, outcomes are likely to be more similar in this group than in others. To account for this, the statistical analysis of the LM outcome data used a multi-level mixed effects model [2]. JtD also allowed the inclusion of couples and stated at the outset that they would be randomised together as in LM; one couple was recruited. The statistical analysis plan detailed the use of a multi-level mixed effects model if > 10 couples had been recruited, with the intervention as a top-level random effect and couples/singles as a lower-level random effect. There are two other potential solutions to this: average the couple’s continuous outcomes and treat them as one individual; or only collect outcome data on one member, the index member. When averaging outcomes across a couple results in a hybrid rather than an individual, the data are difficult to fit in the baseline characteristics table and categorical outcomes cannot be handled in the same way. Indexing is a simple solution, though decisions regarding how to choose the index member from the couple are required and it is wasteful discounting one participant’s data when they are included in the research, especially when recruitment to trials can be difficult.

#### More than one facilitator

More than one facilitator may run a group during the intervention period. Two facilitators delivered LM, REPOSE, STEPWISE and JtD intervention sessions as standard. Additionally, if the group interventions run for more than one session, the facilitator may (and often did) change during the course for a number of reasons. For example, in LM, one facilitator was sick for a number of weeks and two other facilitators covered the group sessions that they missed: four different people (in three combinations of pairs) delivered the intervention to one group of participants. This creates a problem for those wishing to conduct fidelity analyses. In principle, the effect of therapists can be modelled either by using the therapist identifier as a fixed effect in the statistical model or by characterising them in terms of experience. However, where there is more than one therapist per group, it is difficult to identify a therapist effect on an individual participant’s outcome – analysts soon require degrees of freedom which are unavailable from trial samples. Instead, it is common to analyse group interventions using a random effect; doing so does not attempt to explain variation in terms of the participants or the facilitators but rather say that outcomes for individuals in the same group are more similar than for individuals across two different groups. This allows each group (rather than each facilitator) to have different outcomes and acknowledges that facilitators are only one part of this [66]. Nevertheless, the theory of a group effect was not borne out in REPOSE and STEPWISE where the clustering effects were zero.

## Discussion

### Principal findings

#### Participant recruitment and attrition

We have presented the recruitment and attrition rates for our group intervention trials so that future investigators can use these for forecasting recruitment for group intervention trials for similar populations and settings. Recruitment to our group intervention trials was higher than has been reported in individually randomised trials (which may include group interventions) [67], suggesting that recruitment to group intervention trials may be easier than recruitment to individual intervention trials, though comparing recruitment rates across a range of interventions, disease areas and settings is problematic as there are a multitude of factors involved.

A key factor in designing RCTs assessing group interventions is the timing of the various steps required before a participant attends a group session – consent, randomisation and setting dates for the group sessions. There is insufficient evidence from our trials to show that the timing of consent and randomisation affects the rate of attrition before initiation of groups. Attrition before randomisation may be preferred to post-randomisation attrition to maintain statistical power. Delaying randomisation could reduce the time between randomisation and group initiation, therefore reducing the waiting time for participants and the potential for post-randomisation attrition. However, the two trials that delayed randomisation experienced a similar level of post-randomisation attrition to two of the trials that randomised at the point of consent. Attrition also appears unaffected by the point at which the dates for the group sessions are decided, but the timing of setting dates may affect recruitment and attrition in a way not captured by our data. Knowing the dates (or even just the day and time) of the groups before consent could, in theory, reduce recruitment as potential participants may not be able to attend on those dates, but it should in turn reduce attrition after consent as they have already checked their availability.

Delaying randomisation also has implications for capacity of those collecting data as participants may need to be follow-up at the same time.

#### Facilitator training and attrition

Sustaining delivery of group sessions is affected by facilitator attrition and the ability to train new facilitators. We have provided evidence to show that facilitator attrition should be expected for group intervention trials and training sessions should be planned accordingly, throughout the trial. As there are often two facilitators required to deliver group interventions, this may have a bigger impact on group intervention trials than trials assessing individual interventions which usually only have one person delivering the session. Centres attempted to address facilitator attrition and absence, either by having ‘back-up’ facilitators or by training new facilitators. In one case where this was not possible [3, 68], the trial was stopped prematurely.

When designing RCTs of group interventions, consideration should be given to who will be delivering it and how this is funded as this may impact on implementation.

#### Therapeutic dose

Across five trials participants had to attend a median of 62.5% (range 16.7%–80%) sessions, in order to have received a ‘therapeutic dose’; a median of 76.7% (range 42.9%–97.8%) of participants received the ‘therapeutic dose’. These figures can be used to help future investigators determine a per-protocol population for group intervention trials, bearing in mind that this will vary according to the intervention depending on the mechanisms of action. In general, setting the bar low for a therapeutic dose meant that more people received it, though this may influence the effectiveness of the intervention, and should be considered in any process evaluation and analysis.

#### Group size

All studies ran group sessions that were outside the pre-specified ideal size range: across five group interventions, 74.9% of all sessions ran with fewer than ideal numbers and 0.4% ran with more than the ideal numbers. The group intervention aimed at treatment that ran daily for a week was the most successful at meeting the ideal group size; the trial with intervention sessions that were further from the point of randomisation, and further apart in time (booster sessions in STEPWISE), was the least successful and had the lowest average group size. This suggests that the duration of the intervention may be important in maintaining group membership and how many individuals attend all sessions or the number of sessions defining the per-protocol population.

Two trials responded to small group size; one by adding new participants in the second week and one by allowing non-participants to join the groups, which along with merging small groups, are potential solutions to less than ideal group sizes but usage will depend on the intervention and what elements of group processes are important [7].

#### Process evaluation

By nature, group interventions are complex interventions and participants can have different outcomes even if they have received the same intervention delivered by the same facilitator. Process evaluations should be conducted alongside group intervention evaluations to provide information on when the intervention might be successful or when it might fail. Aspects of process evaluation can be assessed at a group or individual level, though guidance assumes interventions work on an individual level. At a group level, quantitative process data, such as non-recruited data and attendance data (recruitment, reach and dose delivered) can be collected, and elements of fidelity, such as treatment receipt and enactment, can be built into quantitative checklists. On an individual level, receipt and enactment can be investigated in participants using qualitative methods.

Some group-specific processes may need a specific group size or for a certain number of sessions to be attended or for certain criteria to be delivered during the sessions. The recently published mechanisms of action in group-based interventions (MAGI) framework [10] may help investigators to identify the group-specific processes essential to the success of a group intervention which should then be used to inform the process evaluation.

#### Clustering issues

We have highlighted two potential issues relating to clustering that may arise in the sample size estimation and the analysis for group interventions: the inclusion of couples and the delivery of the intervention by multiple therapists, which should be accounted for in sample size calculations or in the interpretation of the findings.

### Challenges and solutions for group intervention implementation

Table 8 presents the challenges and potential solutions to the implementation of group interventions in RCTs.

**Table 8:** Challenges and potential solutions to the implementation of group interventions

Challenges in group intervention implementation	Potential solutions
Participant recruitment: A number of participants (likely to be a minimum of 8 in each arm to allow for drop out) need to be recruited before a group intervention can commence Participant recruitment rates can be affected by the relative timing of screening, randomisation and initiation of group, but there is no clear signal as to the best strategy	Recruitment projection and the timing of intervention delivery need to be considered together at the design stage. Those planning group intervention trials should consider demand-forecasting procedures, like those used in clinical settings characterised by surges and slumps and should aim for the maximum group number before commencing the group sessions to allow for attrition. Consider the pros and cons of randomising patients closer to consent (potentially better for accrual rates) or to group initiation (potentially better for retention rates)
Participant attrition: Attrition may be greater where individuals are required to wait longer before starting the group sessions (due to the requirement of recruiting enough people per group)	Delaying randomisation until there are enough recruited participants to run the group may lead to attrition between consent and randomisation but reduce post-randomisation, pre-intervention attrition, which is important to maintain statistical power Maintaining contact with participants before randomisation and the setting up of group sessions may help to reduce pre-randomisation attrition If randomising a number of participants at the same time, trialists should consider and plan for the impact on the follow-up data collection, these participants will need to be followed-up at the same time
Setting group dates: Deciding when to set the group sessions can be challenging. Day/dates can be set before recruitment or once all participants needed for a group session are recruited	Our data did not suggest that either method is superior Those planning group intervention trials should plan groups around the recruitment projection and allow for some flexibility if recruitment does not go as planned
Facilitator training and attrition: Two facilitators are often needed to run group sessions. Recruiting facilitators can be challenging Group facilitators will be lost over the course of the intervention delivery – our data show 70% attrition of trained facilitators in one trial	Allow enough time to recruit and train facilitators prior to the start of recruitment Plan to train replacement facilitators at each site, and/or plan for training sessions throughout the project to account for facilitator attrition or re-training facilitators Group dynamics are important to group interventions; any change in facilitator should be recorded and investigated as part of the process evaluation and through multi-level modelling for analysis where appropriate
Therapeutic dose: This can be difficult to determine for complex interventions but is required for a per-protocol analysis. This may be more difficult for group interventions, as there is less control over what people are exposed to than in one-to-one sessions In our experience, investigators define a therapeutic dose by a threshold number of sessions attended	Defining the per-protocol population should be undertaken by expert consensus, with oversight from the project steering committee. Time should be reserved for this purpose during protocol development For group interventions, ‘therapeutic dose’ may relate to certain intervention criteria being delivered rather than the number of sessions attended and this should be investigated as part of the process evaluation
Group size: An ideal group size will be applicable to the intervention but may be difficult to achieve for all group sessions. Groups may have to run with fewer participants than the ideal. There may be reluctance to amend the group membership (e.g. by adding new participants) once running due to the impact of group dynamics	The impact of group size on the effectiveness of the intervention and must be considered in fidelity assessments and on outcomes Protocol development should include discussions about what happens in the event of small groups and should specify if any number of participants is too few for intervention delivery. Can groups be combined or can new participants or non-participants be added? Consider whether the group size or the maintenance of group dynamics is more important to the intervention
Process evaluation: Assumes interventions work at an individual level meaning some constructs may need adapting for assessing group interventions	Recruitment and ‘dose delivered’ can be assessed at the group level whereas ‘dose received’ can be assessed at the individual level; fidelity can be assessed at the group (delivery) or individual level (receipt and enactment of skills) Process evaluations should include components of the intervention specific to group processes, such as facilitation techniques, group dynamics and development and inter-personal change processes
Clustering issues: Couples recruited to trials and participants that receive the same intervention from the same facilitators are likely to have more similar outcomes than if this was not the case. RCTs may not be powered to use multi-level modelling	This needs to be accounted for in the sample size calculation and made clear when interpreting the findings

*RCT* randomised controlled trial

### Strengths and limitations

The data presented here provide a reference class [44, 69] that researchers can use to plan/manage trials and forecast contingencies. This is valuable as CONSORT diagrams tend to under-report activity before randomisation [47]. Using a case-based approach to explore the experiences of implementing group interventions in trials is appropriate and provides useful data from a range of trials. However, the corpus represents one CTRU’s experience and, while it represents a wide range of clinical and geographic contexts, the settings, roles, interactions and relationships [70] associated with each trial inevitably affect outcomes in ways not captured by our dataset. For instance, the group intervention trials in our sample is weighted towards prevention [2, 3, 5] rather than therapy [1, 4], which are known to have different recruitment dynamics [47], possibly due to motivation to attend and engage [71–73].

### Recommendations

Those planning group intervention trials should consider demand forecasting procedures, as are used in clinical settings characterised by surges and slumps [74–76]. Anecdotal testimony from site staff and trial managers suggests that maintaining contact with participants during recruitment and follow-up stages helps to reduce attrition from research and intervention protocols [49]. Post-randomisation exclusions should be avoided [77] but if randomisation is delayed to reduce the attrition after randomisation [78], then trialists should be aware of the possibility of attrition between consent and randomisation.

Thought should be given to selection and justification of the therapeutic dose and how this may be affected by the number of sessions and group size. As it is unlikely that complex interventions are characterised by linear dose-response patterns [79], trialists should reflect on whether the idea of a ‘therapeutic dose’, proposed by some process evaluators [57], is a useful one. Those retaining session delivery/receipt as an index of ‘therapeutic dose’ should consider how the level at which it is set affects the number of people who will achieve it; the same will be true for fidelity assessment based on satisfying a threshold number of criteria. Guidance on process evaluation [80] currently assumes interventions work at an individual level so constructs may require adaptation in group intervention trials: recruitment and ‘dose delivered’ can be assessed at the group level whereas ‘dose received’ can be assessed at the individual level; fidelity be assessed at the group (delivery) or individual level (receipt and enactment of skills). Recently developed checklists and frameworks [10, 19, 43] for group-based behaviour change interventions can be used to aid the reporting and design of these interventions and for identifying the relevant mechanisms of action, which should inform the associated process evaluation.

As attrition can affect fidelity, study design should include courses of action (group cessation, combination of two groups, membership replenishment, inclusion of non-research participants) for when, inevitably, group sizes drop below an acceptable threshold. As the group context and process are often said to ‘constitute the treatment intervention’ [12], investigators are often reluctant to replenish groups after member attrition, although this is common in many successful ‘open/rolling’ therapy groups [81], including some that have been the subject of trials [50]. Planning for therapist attrition can involve the properly resourced use of contracts, supervision and the training of back-up therapists [50].

Challenges discussed in this paper will vary depending on the population and disease area being studied and the type of group intervention being evaluated and these may be identified in a pilot or feasibility study implementing the intervention.

### Further research

A threat to the implementation of cluster RCTs involving group interventions, not addressed in this paper, is the timing of cluster randomisation. To contain costs, investigators must work to reduce the time between ethical approvals and the set-up of participating centres. Research is needed on how contracting, the allocation of resources, staffing and training (which are not needed at all sites) can be expedited to allow rapid site initiation. Poor group composition due to errors in patient selection can result in disruption of therapy or participant attrition [82, 83]. Further work is required to understand how investigators can employ rational methods of participant allocation to therapy groups [83] in the context of cluster RCTs.

## Conclusions

This paper provides a rational basis for planning group intervention trials, especially how to match the demand of research participants to the supply of trained group facilitators. Investigators need to consider how to time consent and randomisation to minimise post-randomisation attrition. They should plan for both facilitator and participant attrition and consider how group attendance and group size affects treatment fidelity. Further research is needed on expedited set-up of sites in cluster randomised RCTs involving group therapies as well as appropriate baseline group composition and participant replenishment following attrition.

## Data Availability

Requests for patient-level data should be made to the corresponding author and will be considered by all authors who, although specific consent for data sharing was not obtained, will release data on a case-by-case basis following the principles for sharing patient-level data as described by Smith et al. [84]. The presented data do not contain any direct identifiers; we will minimise indirect identifiers and remove free-text data to minimise the risk of identification.

## Abbreviations

CTRU: Clinical Trials Research Unit
IQR: Interquartile range
MRC: Medical Research Council
NHS: National Health Service
NIHR: National Institute for Health Research
NPT: Normalisation Process Theory
R&D: Research & Development
RCT: Randomised controlled trials
ScHARR: School of Health and Related Research
SD: Standard deviation
UKCRC: UK Clinical Research Collaboration
